# Supplementary material for: Photooxidative stress-inducible orange and pink water-soluble astaxanthin-binding proteins in eukaryotic microalga
Source: Commun Biol. 2020 Sep 7;3:490. doi: 10.1038/s42003-020-01206-7 (PMC7477208; doi:10.1038/s42003-020-01206-7)
Supplement: Supplementary file 1 — Supplementary Information [file 42003_2020_1206_MOESM1_ESM.pdf]

Supplementary information

Photooxidative stress-inducible orange and pink water-soluble astaxanthin-binding proteins in eukaryotic microalga

Shinji Kawasaki, Keita Yamazaki, Tohya Nishikata, Taichiro Ishige,

Hiroki Toyoshima and Ami Miyata

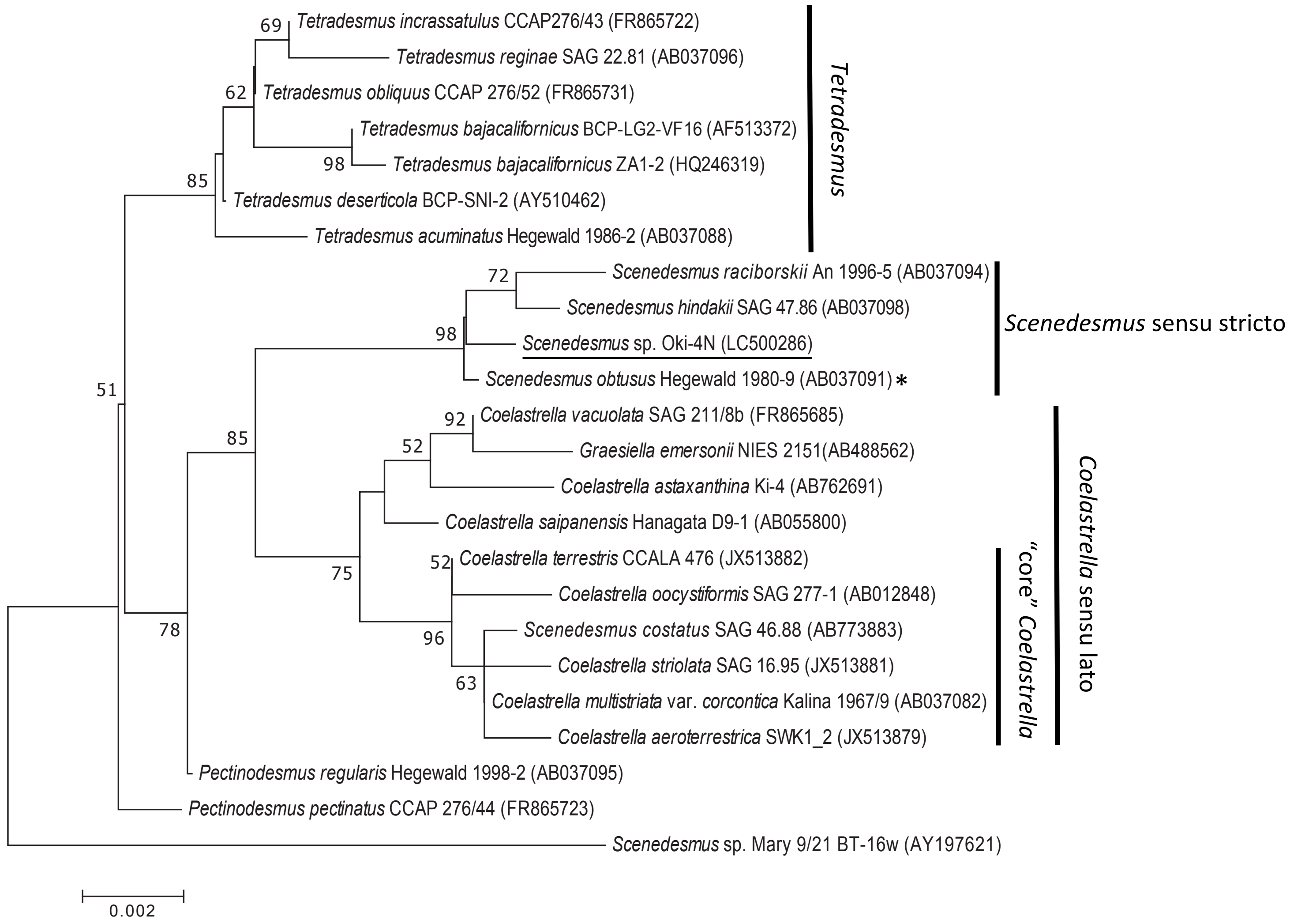

Supplementary Figure 1. Phylogenetic tree of the 18S rRNA gene sequence. Phylogenetic tree of the 18S rRNA gene sequence was constructed using a neighbour-joining (NJ) method using MEGA v. 7.0.2 according to a previous study[24]. Bootstrap values are indicated at the branch points present in >50% of the bootstrap trees. Numbers in parentheses indicate the accession numbers. *Scenedesmus* sp. Oki-4N is underlined. *Scenedesmus obtusus*, a type species of the genus *Scenedesmus*, is asterisked. Scale bar = 0.002 substitutions per site.

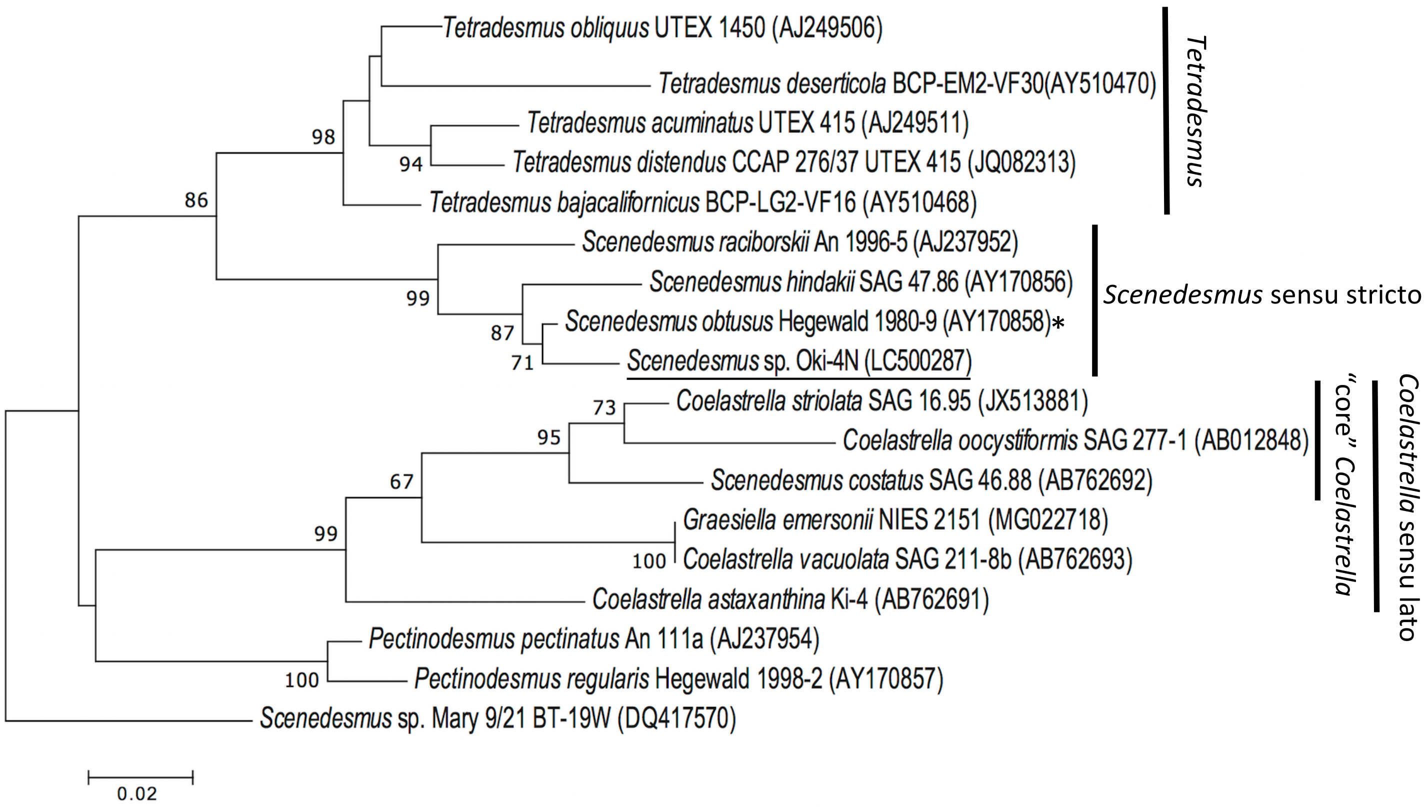

Supplementary Figure 2. Phylogenetic tree of ITS2 gene sequence. Phylogenetic tree of the ITS2 gene sequence was constructed using a neighbour-joining (NJ) methods in MEGA v. 7.0.2 according to a previous study[24]. Bootstrap values are indicated at the branch points present in >50% of the bootstrap trees. Numbers in parentheses indicate the accession numbers. *Scenedesmus* sp. Oki-4N is underlined. *Scenedesmus obtusus*, a type species of the genus *Scenedesmus*, is asterisked. Scale bar = 0.02 substitutions per site.

Supplementary Table 1. Relative peak intensities of each elution peak in Fig. 1d. Peak areas were analysed by LaChrome Elite software (Hitachi).

	Peak area (x 10^4)*		
Stress days	0 day	2 days	4 days
Peak-2	ND	127	230
Peak-3	ND	125	211

ND: Not detected
* Peak area was measured at each peak top.

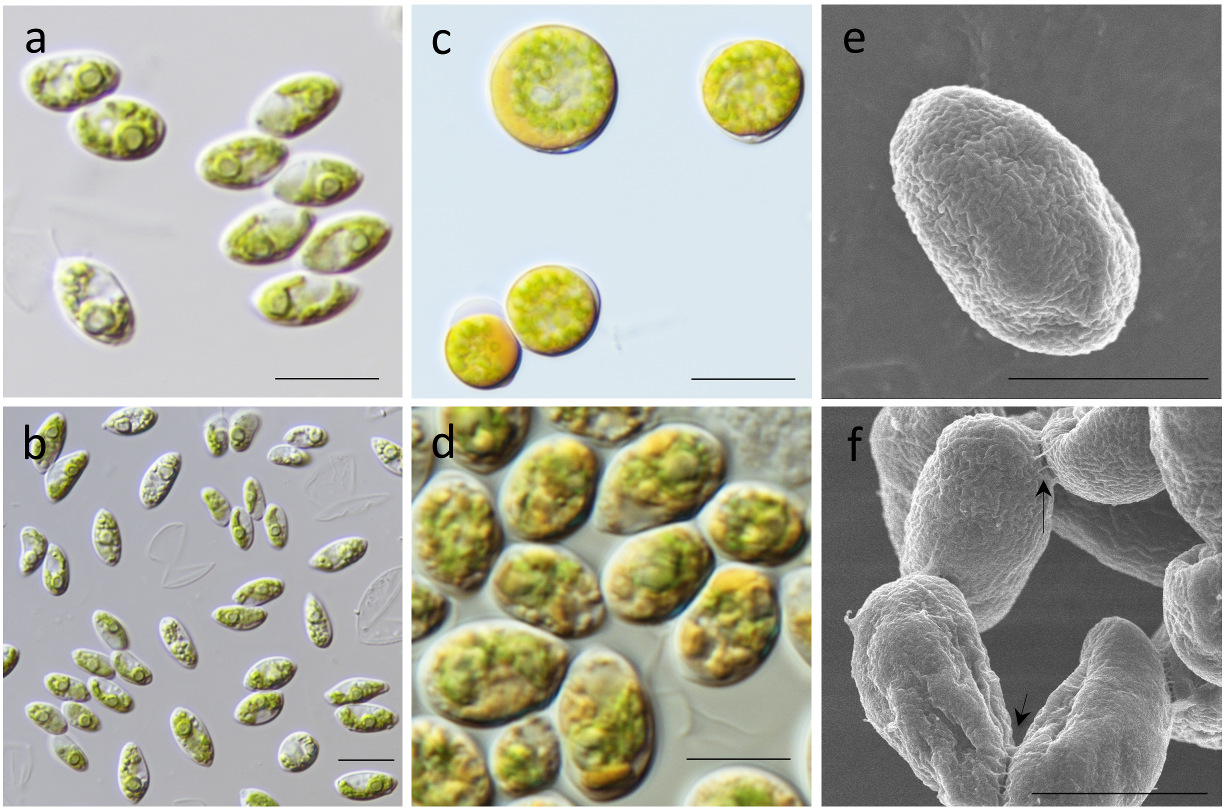

Supplementary Figure 3. Microscopic analyses of OKi-4N. The observed cell features of Oki-4N by optical microscopy **(a-d)** and scanning electron micrographs **(e, f)**. Scale bar = 10 µm. **a, b** Optical microscopic analysis of non-stressed cells revealed that this organism is unicellular or sometimes present in two-, four-, and eight-celled coenobia, oval in shape, and non-flagellar. Each cell contains a pyrenoid, chloroplasts covering the cell sphere, and is uninucleate. **c** Optical microscopy of the cells that were subjected to 0.25 M NaCl w/HL for two weeks. The cells produced large amounts of orangish pigments that covered cell components. **d** Optical microscopy of the cells that were subjected to 0.5 M NaCl w/HL for two weeks. The orangish pigments were localized in the surface portion of the cell or in small vesicles. **e, f** SEM micrographs revealed a smooth cell surface without ornamentations, such as surface ribs which are typically observed in *Coelastrella* species[24]. Arrows indicate fine strands that connect neighbouring cells for the formation of coenobia. Based on genotypical and morphological characterization, the Oki-4N strain was suggested to belong to the genus *Scenedesmus*. Therefore, we tentatively named this strain *Scenedesmus* sp. Oki-4N.

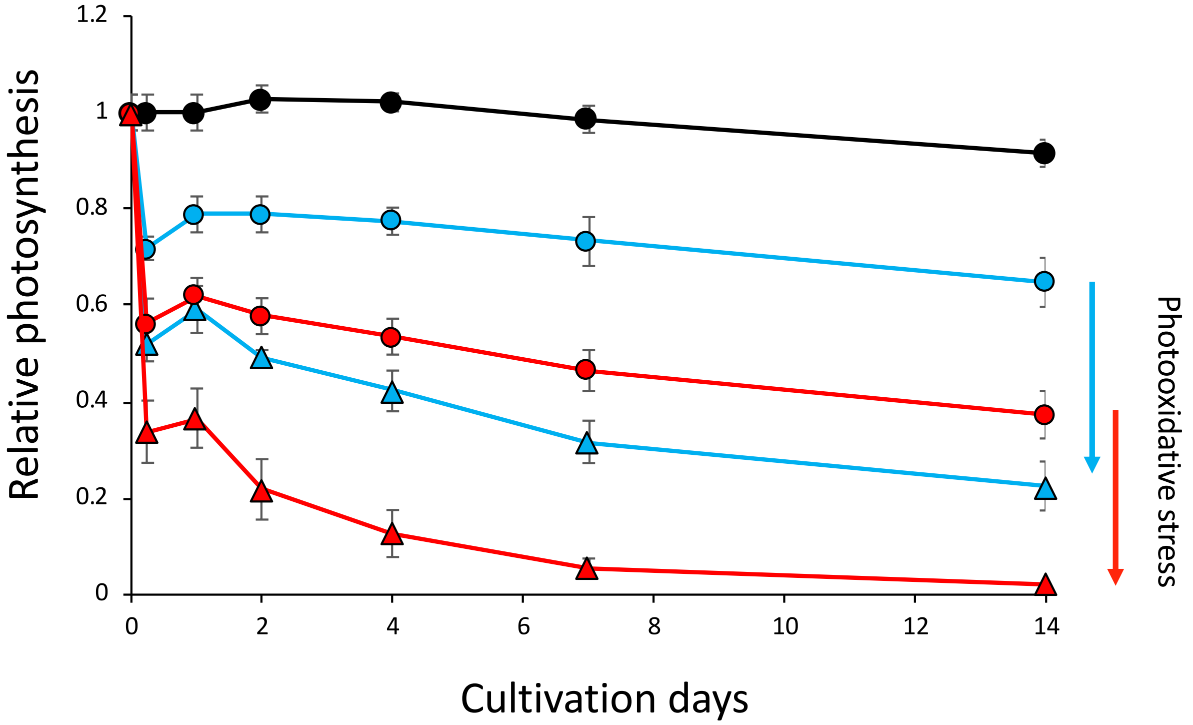

Supplementary Figure 4. Photosynthetic activity of Oki-4N under photo-oxidative stress conditions. Photosynthetic activity was measured by a Clark-type oxygen electrode. Cells were cultivated under low light (LL, 50 µmol m^{-2} s^{-1}) or high light (HL, 800 µmol m^{-2} s^{-1}) conditions with or without salt stress. Photosynthesis was measured for non-stressed cells grown under HL (black circles), 0.25 M NaCl with LL (blue circles) or HL (blue triangles), and 0.5 M NaCl with LL (red circles) or HL (red triangles) and were measured for two weeks after the stress treatments. For each measurement, the cell concentration was adjusted to an OD_{750} value of 1.0. The data represent the average and standard deviation of biological triplicates.

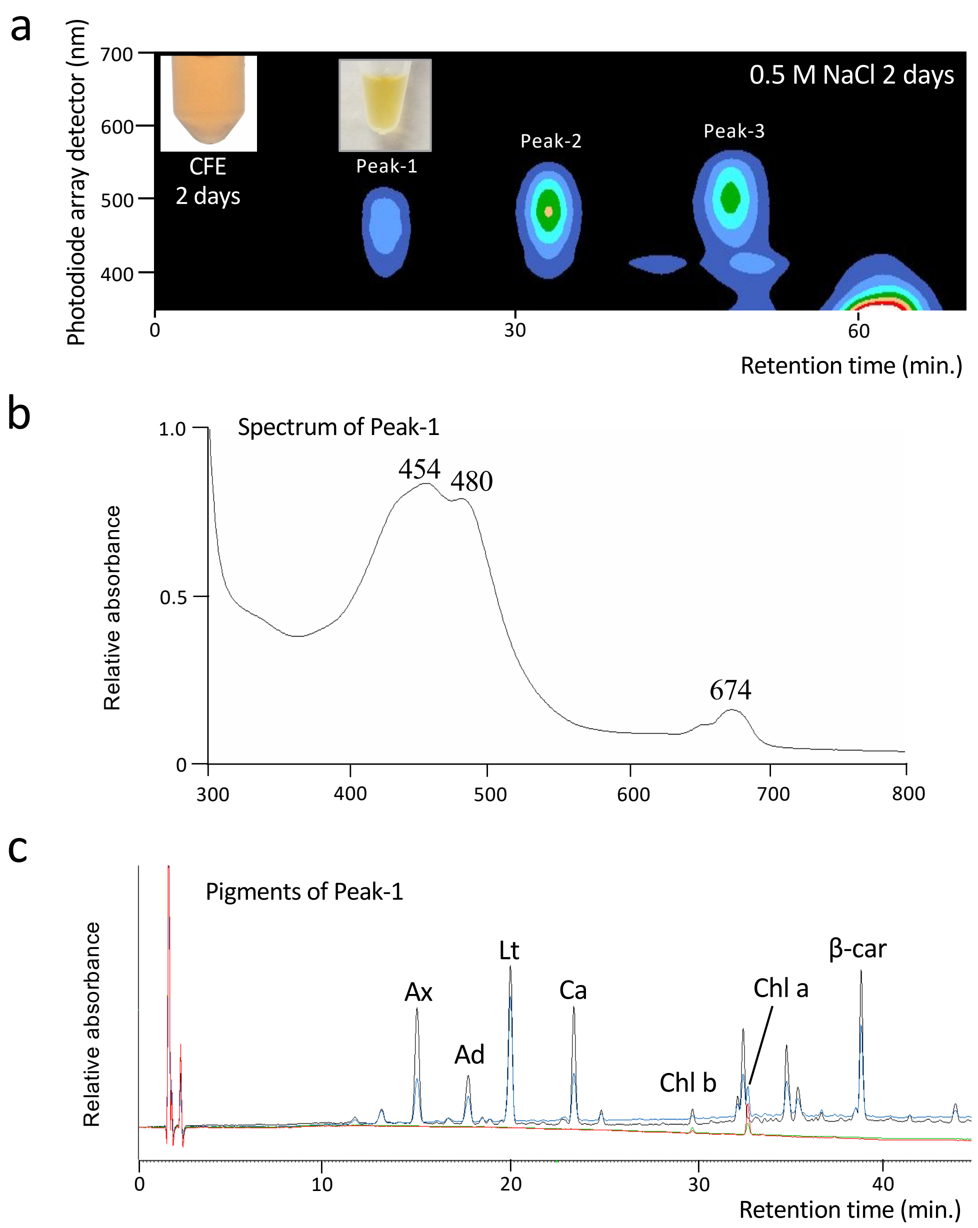

Supplementary Figure 5. Characterization of Peak-1. **a** Elution profiles of gel-filtration column chromatography using salt-stressed (0.5 M NaCl stress for 2 days) Oki-4N cell extracts obtained after ultracentrifugation at 100,000 × *g*. Elution profiles were monitored by using an HPLC photodiode array detector. Pictures of CFE and Peak-1 are shown. **b** Spectrum of Peak-1. **c** HPLC elution profiles of the binding pigments of Peak-1 monitored by 420 nm (blue line), 480 nm (black line), 640 nm (green line), and 660 nm (red line). Pigments were determined based on the absorption spectra obtained using an HPLC photodiode array detector, HPLC retention times, and molecular masses in high resolution liquid chromatography/mass spectrometry analysis compared to standard compounds as described previously[23]. Ax: astaxanthin, Ad: adonixanthin, Lt: lutein, and Ca: canthaxanthin, Chl a: Chlorophyll *a*, Chl b: Chlorophyll *b*, β-car: β-carotene.

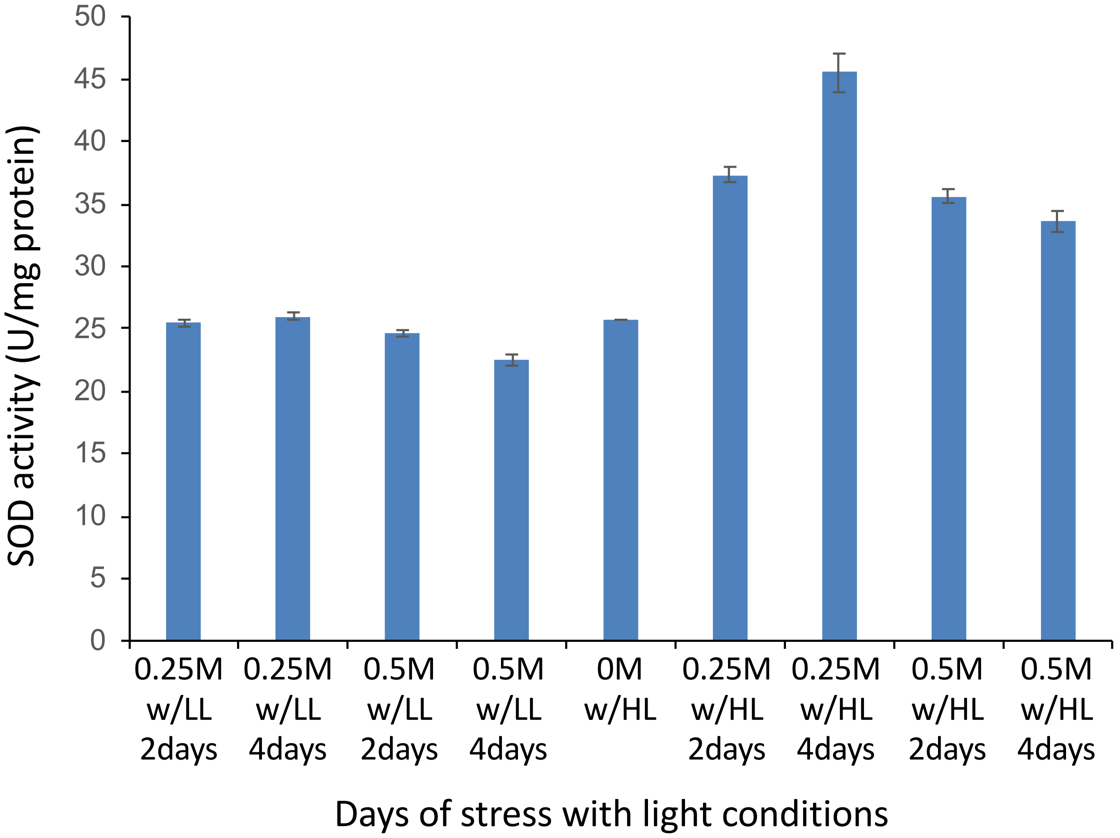

Supplementary Figure 6. Superoxide dismutase activity of cells grown under photooxidative stress conditions. SOD activity was determined by ferricytochrome c-xanthine oxidase method. Cells were cultivated under high light (HL, 800 µmol m^{-2} s^{-1}) conditions. When the cell growth reached OD_{750}=1.0 cm^{-1}, salt stress was started by the addition of sterilized NaCl with low light (LL, 50 µmol m^{-2} s^{-1}) or high light (HL, 800 µmol m^{-2} s^{-1}) conditions. SOD was measured for non-stressed cells grown under HL (0M NaCl with HL, 0M w/HL), 0.25 M NaCl with LL (0.25M w/LL) or HL (0.25M w/HL), and 0.5 M NaCl with LL (0.5M w/LL) or HL (0.5M w/HL) for 2 or 4 days. One unit of SOD activity is defined as the amount of protein that inhibits the rate of cytochrome c reduction by 50%. The data represent the average and standard deviation of triplicate assays.

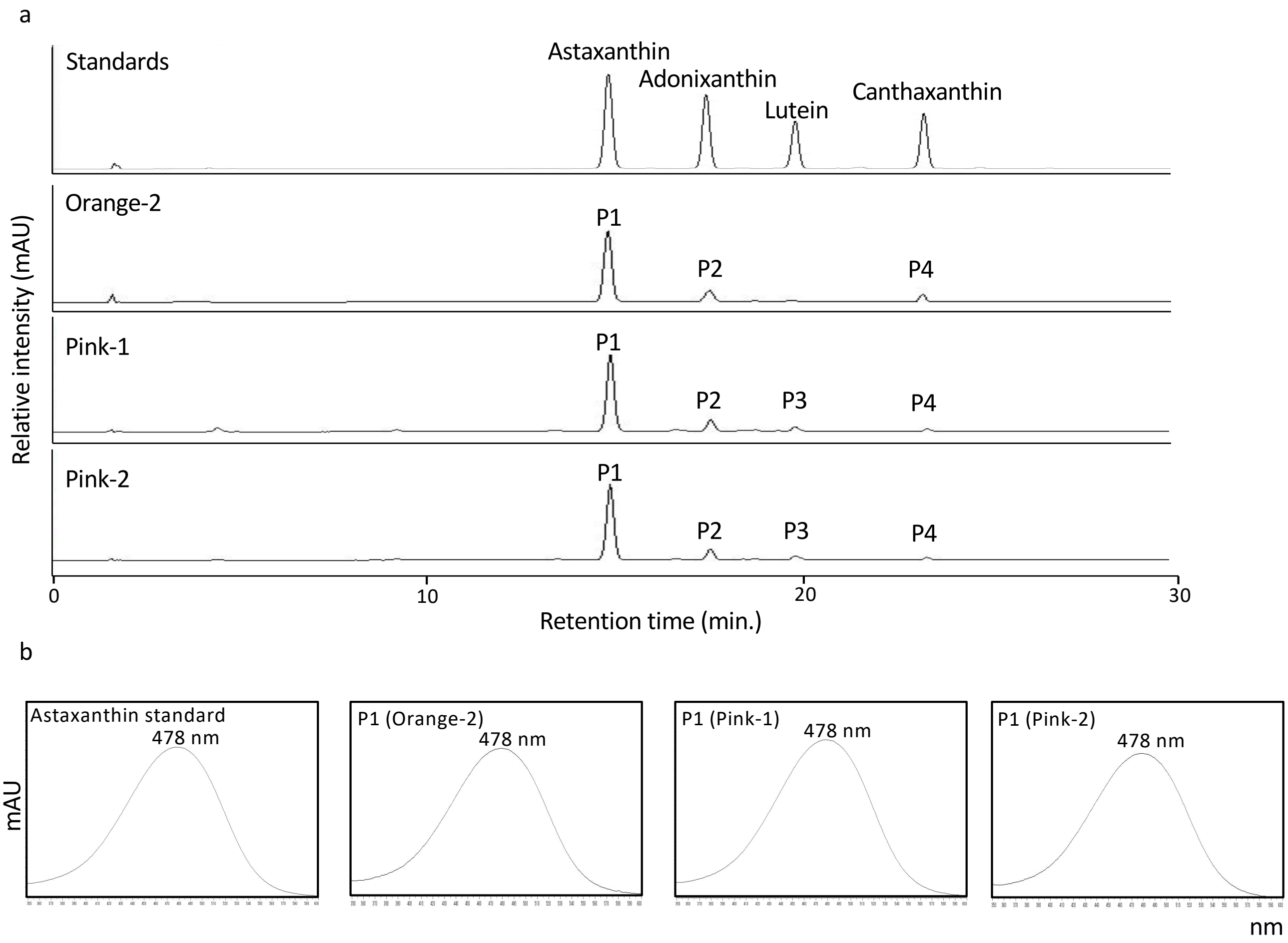

Supplementary Figure 7. Determination of carotenoids bound to Oki-4N AstaP proteins using an HPLC photodiode array detector. **a** Elution profiles of authentic standards and the binding pigments of Oki-4N AstaP-orange2, AstaP-pink1, and AstaP-pink2. The HPLC retention time is shown below the HPLC profiles. **b** Spectrum of an authentic astaxanthin and the P1 pigments from each of the AstaP proteins. Each P1 pigment matched that of the astaxanthin standard with regard to retention times, spectral features, and the *m/z* values obtained from high-resolution LC/MS/MS analysis. The MS-MS spectra of each P1 pigment was matched with that of astaxanthin in the mzCloud database using Compound Discoverer v. 2.1.

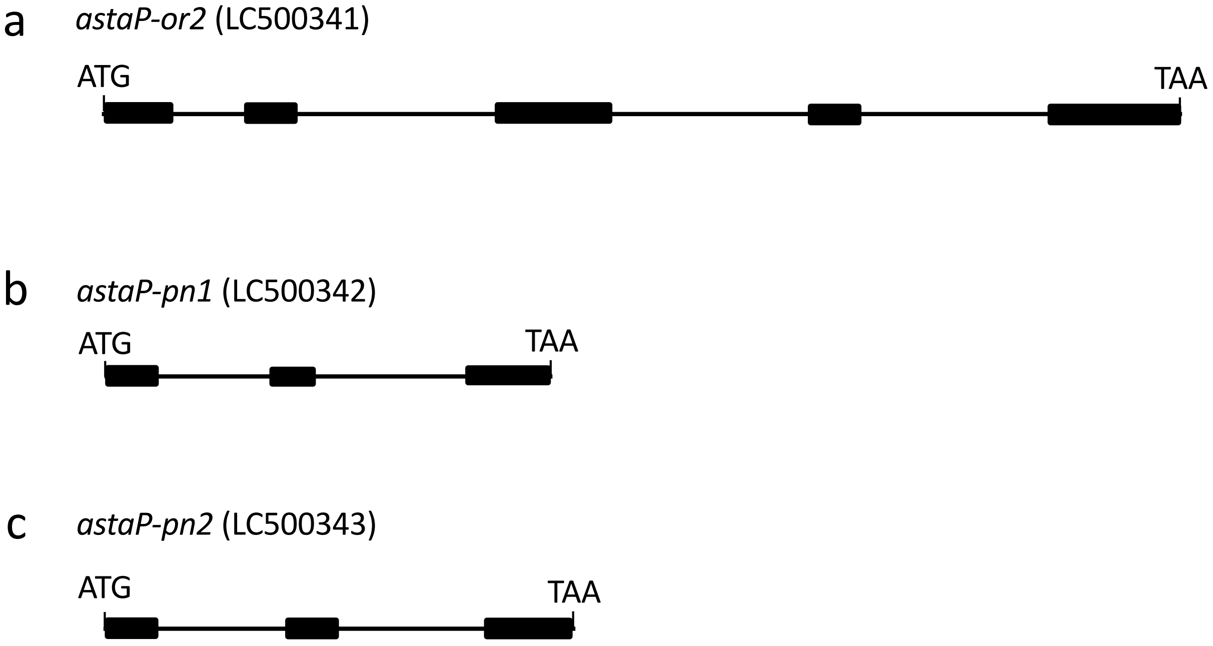

Supplementary Figure 8. **Genome structure of the *astaP* genes.** **a** Genome structure of *astaP-or2* encoding AstaP-orange2. **b** Genome structure of *astaP-pn1* encoding AstaP-pink1. **c** Genome structure of *astaP-pn2* encoding AstaP-pink2. The start and stop codons are indicated. Filled boxes represent exons, and the lines between filled boxes represent introns. Numbers in parentheses indicate the accession numbers.

a

```
A-pink1     MKSFISLCVLGCLFSAALGRQLQQAPAPAKRGLTAALQQAAASAPQLSTLVAAIQASGLQ 60
A-pink2     MKSFIALCVLGCLFGAAFGRQLQQAPAAASGGLTAALQQAAASAPQLSTLVAAIQASGLQ 60
            *****:********.**:*********.*. *****************************

A-pink1     IPDDAAWTIFAPTNEAFADDDVREKTGLTAQQLLKPANKDALVKLLSYHVVPAGAVRSTK 120
A-pink2     IPDDAAWTIFAPTNEAFADDDVREETGLTAHQLLEPANRDALVQLLSYHVVPAGAVSSSQ 120
            ************************:*****:***:***:****:************ *::

A-pink1     LTDGQVLQTLLKGATLKVDLDEDDGRRKIEIESSAGDDDGADVVRADIVAGNSIIHVVDD 180
A-pink2     LTEGQVLQTLLEGATLKVDLDEDDGRREIEIEATAGDDDGADVVTADIVAGNSIIHVVDD 180
            **:********:***************:****::********** ***************

A-pink1     VLIPAALRKSG 191
A-pink2     VLIPAALRRSG 191
            ********:**
```

b

```
A-orange1(Ki-4) MRRNSIVLLVLFCLVALATAATPKANATTAKPASTTSTPVYATLSNAVTAGAAAPQLTTL 60
A-pink1(Oki-4N) -MKSFISLCVLGCLFSAALGRQLQQAPAPAKRGLT------AALQ---QAAASAPQLSTL 50
                  :. * * ** **.: * .   :  .:.** . *      *:*.    *.*:****:**

A-orange1(Ki-4) FAAVRAANVTGALTANTTWTILAPTNDAFAKR--LAKLNLTADAVLK--NKDLLVKILSY 116
A-pink1(Oki-4N) VAAIQASGLQ--IPDDAAWTIFAPTNEAFADDDVREKTGLTAQQLLKPANKDALVKLLSY 108
                .**::*:.:   :. :::***:****:***.     * .***: :**  *** ***:***

A-orange1(Ki-4) HVIPSGAVYSKALKDNATVATALKDASVTVRLYQG----KVMFKGPVN---KAQVTVADI 169
A-pink1(Oki-4N) HVVPAGAVRSTKLTDGQVLQTLLKGATLKVDLDEDDGRRKIEIESSAGDDDGADVVRADI 168
                **:*:*** *. *.*. .: * **.*::.* * :.    *: ::....    *:*. ***

A-orange1(Ki-4) KAGGSVIHVINDVLLPPGVVSDAVAKQWKAEWEAMKAEKKVAPKATTGRRFLLF 223
A-pink1(Oki-4N) VAGNSIIHVVDDVLIPAALRKSG------------------------------- 191
                 **.*:***::***:*..: ...
```

Supplementary Figure 9. Comparison of amino acid sequences among AstaP-proteins. **a** Amino acid sequence similarity between AstaP-pink1 (A-pink1) and AstaP-pink2 (A-Pink2). AstaP-pink1 and AstaP-pink2 showed 89.5% identity. **b** Amino acid sequence similarity between AstaP-orange1 (A-orange1 from *C. astaxanthina* Ki-4) and AstaP-pink1 (A-Pink1 from *Scenedesmus* sp. OKi-4N). AstaP-pink1 showed 41.4% and 41.9% identity to that of AstaP-orange1 and AstaP-orange2, respectively, in the overlapping regions. Identical amino acid residues are indicated by asterisks, and similar amino acid residues are indicated by dots. N-terminal signal peptides are shown in green fonts. Potential sites for N-linked glycosylation are shown in red fonts. H1 and H2 motifs of the fasciclin domains are shown in boldface italics.

a. 1-213 region of Oki-4N AstaP-orange2 vs. full length Ki-4 AstaP-orange1

```
AstaP-orange1   MRRNSIVLLVLFCLVALATAATPKANATTAKPASTTSTPVYATLSNAVTAG-AAAP-QLT 58
AstaP-orange2   -MQAMKAILLLACCFAVARAQT-----------------TFPSLVDAVAAANASAPQQMS 42
                  :   .:*:* * .*:* * *                 .:.:* :**:*. *:** *::

AstaP-orange1   TLFAAVRAANVTGALTANTTWTILAPTNDAFAKRLAK-LNLTADAVLK--NKDLLVKILS 115
AstaP-orange2   ILLAAVQAAGVAGSLGPNTTWTILAPTNNAFVSRLNESLGITPQELLLPENRDVLVEVLS 102
                 *:***:**.*:*:* .***********:**..** : *.:*.: :*   *:*:**::**

AstaP-orange1   YHVIPSGAVYSKALKDNATVATALKDAS-VTVRLYQGKVMFKGPVNKAQVTVADIKAGGS 174
AstaP-orange2   YHVIPSGAVLSSQLTDGQEAPTALAEAAPLTVSIANGNVTFEGANNNAAVTTADIEAGSS 162
                ********* *. *.*.  ..*** :*: :** : :*:* *:*. *:* **.***:**.*

AstaP-orange1   VIHVINDVLLPPGV--VSDAVAKQWKAEWEAMKAEKKVAPKATTGRRFLLF 223
AstaP-orange2   VIHIIDDVLLPAGIGNFPNATNTTGPVVYESIAAALEAANGTNSSLSILLA 213
                ***:*:*****.*:  ..:*. .   . :*:: *  :.*  :.:.  :**
```

b. 179-402 region of Oki-4N AstaP-orange2 vs. full length Ki-4 AstaP-orange1

```
AstaP-orange1   MRRNSIVLLVLFCLVALATAATPKANATTAKPASTTSTPVYATLSNAVTAG-AAAPQLTT 59
AstaP-orange2   --------------------- NFPNATN-----TTGPVVYESIAAALEAANGTNSSLSI 210
                                         ***      **.. ** ::: *: *. .: ..*:
AstaP-orange1   LFAAVRAANVTGALTANTTWTILAPTNDAFAKRLAK-LNLTADAVLK--NKDLLVKILSY 116
AstaP-orange2   LLAAVEAAGVGANLTNDTAWTILAPTNDAFVTRLNDSVGITPEQLLLPENRDTLVQVLSY 270
                *:***.**.* . ** :*:***********..** . :.:*.: :*   *:* **::***

AstaP-orange1   HVIPSGAVYSKALKDNATVATALKDAS-VTVRLYQGKVMFKGPVNKAQVTVADIKAGGSV 175
AstaP-orange2   QVIPSGAVLSSQLTDGQEAPTALAEAAPLTVSIANGTVTFVGGNSNASVTTADIQAGASV 330
                :******* *. *.*.  ..*** :*: :** : :*.* * *  .:*.**.***:**.**

AstaP-orange1   IHVINDVLLPPGVVSDAVAKQWKAEWEAMKAEKKVAPKATTG---RR-----------FL 221
AstaP-orange2   IHVIDDVLLPAGVGTPEVVPTVDALQGAAAPATAAAPAVPAGPAQRQTSGASTTVSSVFW 390
                ****:*****.** :  *.   .*   *  . . .** ..:*   *:           *

AstaP-orange1   LF---------- 223
AstaP-orange2   MGAALAIANALL 402
                :
```

Supplementary Figure 10. Comparison of amino acid sequence among AstaP proteins. **a** Amino acid sequence similarity between AstaP-orange1 (*C. astaxanthina* Ki-4) and the front portion of AstaP-orange2 (1-213 residues in 402 amino acids of AstaP orange2 from *Scenedesmus* sp. OKi-4N). The front part of AstaP-orange2 showed 43.1% identity to AstaP-orange1 in the overlapping region. **b** Amino acid sequence similarity between AstaP-orange1 from *C. astaxanthina* Ki-4 and the rear part of AstaP-orange2 (179-402 residues in 402 amino acids of AstaP-orange2 from *Scenedesmus* sp. OKi-4N). N-terminal signal peptides are shown in green fonts. Potential sites for N-linked glycosylation are shown in red fonts. The H1 and H2 motifs of the fasciclin domains are shown in boldface italics. A predicted C-terminal hydrophobic GPI anchor signal sequence was underlined, and potential (yellow font) and alternative (orange font) GPI-modification sites are shown.

Supplementary References

23. Kawasaki, S., Mizuguchi, K., Sato, M., Kono, T. & Shimizu, H. A novel astaxanthin-binding photooxidative stress-inducible aqueous carotenoprotein from a eukaryotic microalga isolated from asphalt in midsummer. *Plant Cell Physiol.* **54**, 1027–1040 (2013).
24. Kawasaki, S., Yoshida, R., Ohkoshi, K. & Toyoshima, H. *Coelastrella astaxanthina* sp. nov. (Sphaeropleales, Chlorophyceae), a novel microalga isolated from an asphalt surface in midsummer in Japan. *Phycol. Res.* **68,** 107-114 (2020).
