# Supplementary material for: Photooxidative stress-inducible orange and pink water-soluble astaxanthin-binding proteins in eukaryotic microalga
Source: Commun Biol. 2020 Sep 7;3:490. doi: 10.1038/s42003-020-01206-7 (PMC7477208; doi:10.1038/s42003-020-01206-7)
Supplement: Supplementary file 2 — Description of Additional Supplementary Files [file 42003_2020_1206_MOESM2_ESM.pdf]

Description of Additional Supplementary Files

File Name: Supplementary Data 1
Description: Full size images of Northern-blot analysis shown in Fig. 4a.

File Name: Supplementary Data 2
Description: Raw data for the enzymatic activity assay shown in Fig. 4b.
