# Supplementary figures and images for: Photooxidative stress-inducible orange and pink water-soluble astaxanthin-binding proteins in eukaryotic microalga
Source: Commun Biol. 2020 Sep 7;3:490. doi: 10.1038/s42003-020-01206-7 (PMC7477208; doi:10.1038/s42003-020-01206-7)

*astaP-pn1*  
(*astaP-pn2*)

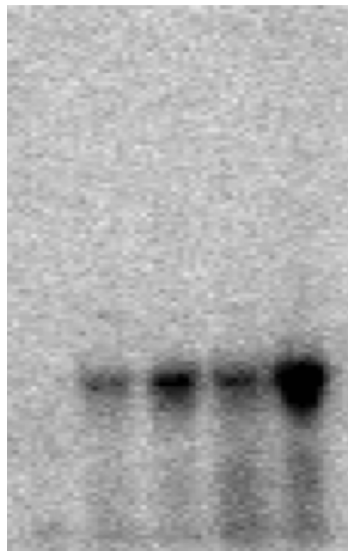

— 1.0kb

*astaP-or2*

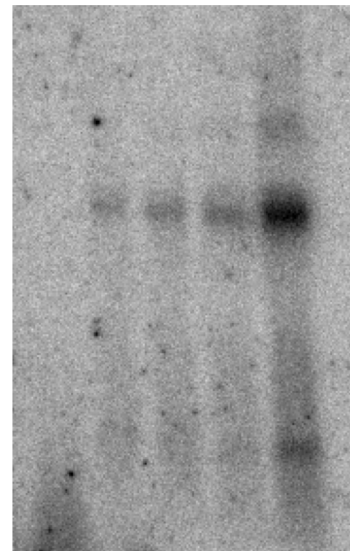

— 1.5kb

Supplement: Supplementary file 3 — Supplementary Data 1 [file 42003_2020_1206_MOESM3_ESM.pdf]
