# Supplementary material for: Photooxidative stress-inducible orange and pink water-soluble astaxanthin-binding proteins in eukaryotic microalga
Source: Commun Biol. 2020 Sep 7;3:490. doi: 10.1038/s42003-020-01206-7 (PMC7477208; doi:10.1038/s42003-020-01206-7)
Supplement: Supplementary file 4 — Supplementary Data 2 [file 42003_2020_1206_MOESM4_ESM.pdf]

Supplementary data for Fig. 4b

n	AstaP-pink1	AstaP-pink2	AstaP-orange2	NaN3 0.5mM	NaN3 1.0mM
1	44.8	37.1	35.2	20.6	43.0
2	41.9	39.3	36.8	19.1	40.6
3	42.9	35.8	29.8	22.2	40.8
mean	43.2	37.4	33.9	20.6	41.5
SD	1.47	1.77	3.67	1.55	1.33
